# Effect of proteolytic activities in combination with the pectolytic activities on extractability of the fat and phenolic compounds from olives

**DOI:** 10.1186/s40064-016-2367-2

**Published:** 2016-06-16

**Authors:** Youssef Moustakime, Zakaria Hazzoumi, Khalid Amrani Joutei

**Affiliations:** Laboratory of Bioactive Molecules: Structure and Function, Faculty of Science and Technology Fez, B.P. 2202, Road of Imouzzer, Fez, Morocco

**Keywords:** Moroccan Picholine, Olive oil, Proteolytic enzyme, Pectolytic enzyme, Extractability, Phenolic compounds, Ripening, Pectins

## Abstract

During the extraction, a portion of oil remains trapped inside the cells and its release requires the degradation of the walls and cell membranes, especially when the fruits have not reached a maximum maturity which is likely to cause an optimal embrittlement of the parietal structures and cell membrane. This can be done by specific enzymes necessary for the degradation of various cellular barriers. Three different enzyme treatments proteolytic, pectolytic or both are applied on the Moroccan Picholine olives from veraison to maturity of the fruit. The effect of these treatments is evaluated by olive oil diffusion, its phenolic content (PC) and cellular embrittlement determination of olives during ripening. The pectolytic activities lead to a significant increase in both the oil extractability (76 % at veraison and 14 % at maturity) and the PC (up to 50 % of gain compared to the control at veraison and 27 % at maturity). The proteolytic activities applied alone have no significant effect on the extractability and the polyphenols levels of oils. Furthermore, when these proteolytic activities are added in combination with the pectolytic activities, the oil extractability is doubled at veraison and its flowing up to 99 % at maturity that barely 84 % in the control in addition to a richness of polyphenols which can reach 84 % more compared to the control. This increase in polyphenols wealth is probably due to the degradation of cell walls, cellular and vacuolar membranes by enzyme activities releasing PCs that were previously associated with these structures in the drupe.

## Background

The beneficial effects of olive oil on human health are due to both its high content of monounsaturated fatty acids and antioxidants (Visioli and Galli 1998). During extraction, the change in content of some components depending essentially on the techniques used (Morales and Aparicio 1999; Amirante et al. 2001), with the appearance of new components following chemical and/or enzymatic pathways (Ranalli et al. 1999). Many factors, such as the mixing temperature, the exposure time of the olive paste in contact with air (Servilli et al. 2003), or the use of enzymes (Vierhuis et al. 2001), can significantly influence the yield and quality of the oil.

The mesocarp (the flesh) and the pericarp (the skin) contain 96–98 % of the olive oil and only 2–4 % is in the endocarp (pit). During extraction, a portion of oil remains trapped inside the cells and release requires the degradation of the walls and cell membranes, especially when the fruits have not reached an optimal fragility. This can be done by specific enzymes necessary for degradation of various cellular barriers. Enzymes present in the fruit of the olive are generally made ineffective during the process of extracting oil or grinding step because of their inhibition by phenolics compounds present in the cells. Furthermore, many studies have been conducted on the effects of exogenous enzymes on the extraction and the characteristics of the olive oil (Domínguez et al. 1994; Garcia et al. 2001; Ranalli and De Mattia 1997, 1996; Vierhuis et al. 2001, 2003). Most of these works relate only pectolytic activities while proteolytic activities have been very little studied.

The aim of this work was to study the extractability of olive oil and the diffusion of phenolic compounds in fat matter in order to increase oil yields with good phénolic quality. This by performing of exogenous proteolytic and pectolytic enzymes applied on pitted olives at different stages of ripening. The effects of these treatments were evaluated by the yields of extracted oils and their flow percentages of the total fat content of the olives and the evolution of their total polyphenol contents during the ripening period. The weakening of the structures of fruit estimated by changes in content of protopectines cell walls and endogenous enzyme activities during ripening olives was also determined.

## Methods

### Plant material

Olives, Moroccan Picholine variety rainfed, were manually harvested all around olive trees who grown in the botanical garden of the Faculty of Science and Technology of Fes—Morocco-. Five samples were taken during the period of maturation of the drupe with four distinctive stages according to the color of the drupe: green fruits (first sampling), fruit with small reddish spots (second sample), fruit turning colors and purple fruits (3rd and 4th samples) and completely black fruit (5th sampling).

For each sample, three enzymatic treatments are performed on pitted olives: (1) Treatment with a proteolytic enzyme [4 grams/hectoliter (g/hl)] (2) Treatment with a pectolytic enzyme (4 g/hl) (3) Treatment that combines the previous two enzymes (4 g/hl for each enzyme). These enzymes are commercial preparations containing a number of specific activities for the degradation of the membrane proteins and polysaccharides which ensure the rigidity of the cell wall. Pitted olives under the same experimental conditions without the addition of enzyme activities constitute the control.

## Moisture determination olives

Sample moisture was determined by drying olive fruits in an olive stove at 105 °C according to the UNE Standard Spanish Method (Aenor 1973). A 40 g-sample is placed into a porcelain capsule and dried in a stove at 105 °C for 6 h. Subsequently, the sample was cooled in a desiccator, weighted and reintroduced into the oven. This operation was repeated until variations in Moisture weight loss and volatile loss were <0.02 g.

## Determination of the total oil content

The extraction of the total oil content is carried out using hexane in a Soxhlet extractor for 4 h from the dryer used for the determination of humidity content material (Aenor 1961). After that, the traces of solvent are removed by evaporation. The determination of the oil content is determined by the weight of dry matter (%dry weight), and the weight of wet matter (%wet weight).

## Extraction and determination of phenolic compounds (PC)

The procedure of phenolic compounds extraction from olive fruit is based on the method of Brenes et al. (1995), with some changes. Olive pulp (10 g) is mixed with 30 ml of methanol: water 80/20 (v/v). The mixture was centrifuged for 5 min at 3500 rpm and then filtered. This extraction was repeated three times. The extracts are collected after evaporation of the organic solvent. Phenolic compounds are extracted with ethyl acetate (5 × 20 ml). The extraction of oil polyphenols is performed with methanol according to the method of Vázquez-Roncero et al. (1973).

The assay of total polyphenols is based on the reduction of acid phoshomolybdique of Folin-Ciocalteu agent by polyphenols in alkaline medium (Catalano et al. 1999).

## Isolation and determination of total polysaccharides

The isolation of pectins is achieved from 50 g-olive pulp according to the procedure of Saulnier and Thibault (1987) which implements the precipitation of insoluble material in alcohol (MIA) by several washings with ethanol 95°. From this MIA, successive extractions with water, Na oxalate, hydrochloric acid and soda are done.

The assay principle of the pectic substances is based on the colorimetric determination of the galacturonic acid content of the pectic chains hydrolysed in hot acidic medium of different fractions isolated in the presence of 3-hydroxydiphenyl (Robertson 1979).

The three enzymatic treatments mentioned above are used on the MIA obtained from the last sample in order to highlight the presence of phenolic compounds bound to the cell wall.

## Extraction and measurement of endogenous enzyme activities

1 g of olive pulp homogenized for 30 s in 25 ml potassium phosphate buffer (0.05 M, pH 6.6) with 0.2 g of Triton X-100 using a Polytron homogenizer. 25 mg Polyvinylpyrrolidone (PVPP) were added and the suspension was centrifuged at 4 °C for 15 min at 13,000 rpm. The supernatant is filtered through glass wool and used as a source of crude enzyme (Jesús Tovar et al. 2002). The various activities are determined as follows:

The polygalacturonase (PG) by the method of Nelson (1944).

The pectinestérases enzymes (PE) are assayed by the method of Baron (1984).

### Statistical analyses

An analysis of variance was performed for each parameter studied. The multiple comparison test averages Tukey post hoc is used to test for significant differences between treatments (at 5 %). Univariate analysis was used to test for significant differences in treatment, their interaction for a single parameter. All statistical analyzes were performed with IBM. SPSS statistics, version19. The results of each experiment are made of triplets.

## Results and discussion

### Study of the oil extractability and its PC content during the ripening of olives

During ripening olives, fat content increased on a regular basis since veraison until full maturity (Table 1). This increase is due to the increase of the weight and the diameter and the decrease of the humidity of the olives. The extractability of oils expressed by the % of diffusion of the oils relative to the total content in the olives is very low in the ripening and increases during the maturation to 84 % in maturity. Moreover, total polyphenol contents they know fluctuations during the ripening of olives, these contents are high at veraison, a decrease importantly in the ripening and increasing again at maturity. However, despite relatively high CP content in olives, the concentration of oil in these molecules decreases very importantly during ripening.

**Table 1:** Agronomic indices of olives during maturation

Harvest date	18 Oct. 2013	04 Nov. 2013	20 Nov. 2013	06 Déc. 2013	20 Déc. 2013
Weight of 100 drupes (g)	309 ± 11.4	327 ± 8.4	364 ± 7.6	399 ± 7.9	439 ± 8.2
Diameter (cm)	3.6 ± 0.8	3.62 ± 0.8	3.63 ± 0.7	3.96 ± 0.8	4.32 ± 0.7
Humidity (%)	63.8 ± 0.2	63 ± 0.9	63 ± 0.9	58 ± 0.7	51 ± 0.4
Olive oil content (g/100 g olive flesh)	2.5 ± 0.1	3.2 ± 0.24	3.7 ± 0.3	7.1 ± 0.2	14.4 ± 0.3
Oil content extracted (g/100 g olive flesh)	0.62 ± 0.1	1.13 ± 0.1	2.7 ± 0.1	5.43 ± 0.3	12.13 ± 0.2
% of oil diffusions/total content	25.1 ± 1	35.6 ± 1.6	72.3 ± 1.2	76.5 ± 4.4	84.2 ± 1.1
Content of PC in olive flesh (mg/g)	73.7 ± 1.3	77.2 ± 6.7	58.4 ± 1.8	58.9 ± 2.4	66.4 ± 6.8
PC concentration of oil (%)	0.9 ± 0.04	0.6 ± 0.04	0.53 ± 0.05	0.4 ± 0.02	0.3 ± 0.05

## Influence of proteolytic and pectolytic activities on the extraction of oil during the ripening of the olives

The quantities of oil extracted undergo a steady increase during ripening (Table 2), reaching a maximum at maturity for both the control, and for olives processed by enzymatic activities. The proteolytic activities have no influence on extractability when the olives are not mature completions; barely 8 % improvement is recorded at full maturity. Contrariwise, the pectolytic activities lead to a significant improvement in oil extractability from the first samples when the olives are being veraison. This improvement can reach 76 % at veraison and gradually decreases to 14 % at maturity. In addition, when the proteolytic activity is added in combination with pectolytic activities, there is a significant improvement in oil extractability begins from veraison except the last sample whose improvement is not significant compared to pectolytique treatment. This improvement reached 92 % at veraison and 17.5 % at maturity.

**Table 2:** Evolution of oil contents extracted by pectolytic and proteolytic enzyme treatments during the olives ripening and their relative flowing percentages to the control

Sample	Lipids extracted (g/100 g olive flesh)				% Flowing compared with the control		
	C	Prot	Pect	C.T	Prot	Pect	C.T
S1 (18/10)	0.62 ± 0.03^a^	0.65 ± 0.03^a^	1.09 ± 0.04^b^	1.2 ± 0.04^c^	5.91 ± 4.05^a^	75.26 ± 6.71^b^	92.26 ± 6.87^c^
S2 (04/11)	1.13 ± 0.04^a^	1.22 ± 0.02^a^	1.56 ± 0.05^b^	1.74 ± 0.06^c^	8.14 ± 1.79^a^	38.05 ± 4.68^b^	54.28 ± 5.34^c^
S3 (20/11)	2.7 ± 0.05^a^	2.72 ± 0.06^a^	3.4 ± 0.1^b^	3.65 ± 0.05^c^	1.23 ± 2.13^a^	25.43 ± 3.8^b^	35.18 ± 1.85^c^
S4 (06/12)	5.43 ± 0.31^a^	5.6 ± 0.22^a^	6.2 ± 0.1^b^	6.86 ± 0.03^c^	3.19 ± 3.42^a^	14.3 ± 1.66^b^	26.45 ± 0.56^c^
S5 (20/12)	12.13 ± 0.15^a^	13.1 ± 0.46^b^	13.9 ± 0.1^c^	14.25 ± 0.05^c^	8 ± 3.78^a^	14.73 ± 0.85^b^	17.5 ± 0.42^b^

*C* control, *Prot* proteolytic treatment, *Pect* pectolytic treatment, *C.T* Combined treatment

For each sample, the values followed by different letters are significantly different (P = 0.05)

Moreover, in the industrial extraction phases, olives do not release all of their oil, much remains locked inside the pomace cells. Table 3 shows the flowing percentages of fat olive cells during ripening and we find that without any enzymatic activity, cells olives release only 25 % of oil at veraison, this percentage increases during ripening to reach only 84 % at maturity. The use of proteolytic activities has no influence on the extractability when the olives are not mature completions. At maturity, the quantities of oil extracted do not exceed 90 % of the total fat olives and this content is not significantly different compared to the quantities extracted from the control. The pectolytic activities on them enhance such extractability since at veraison, the contents of which diffuse oils already reached 44 % and these values continue to increase to around 97 % at maturity. Contrariwise, when the pectolytic activities are added in combination with proteolytic activity, there is a very significant improvement in oil extractability begins from veraison except the last sample whose improvement is not significant compared to pectolytique treatment. This improvement can double at veraison compared to the control (48 %) and can reach up to 99 % at maturity.

**Table 3:** Evolution of oil flowing percentages by proteolytic and pectolytic enzyme treatments during the olives ripening

Sample	Total lipids (g/100 g olive flesh)	% Flowing			
		C	Prot	Pect	C.T
S1 (18/10)	2.5 ± 0.1	25.14 ± 1.01^a^	26.47 ± 1.02^a^	43.83 ± 1.64^b^	48.13 ± 1.6^c^
S2 (04/11)	3.2 ± 0.24	35.63 ± 1.64^a^	38.23 ± 0.65^a^	48.76 ± 1.64^b^	54.5 ± 1.9^c^
S3 (20/11)	3.7 ± 0.33	72.3 ± 1.23^a^	73.12 ± 1.74^a^	90.8 ± 2.73^b^	97.85 ± 1.35^c^
S4 (06/12)	7.1 ± 0.21	76.52 ± 4.43^a^	78.6 ± 3.1^a^	87.4 ± 1.25^b^	96.7 ± 0.46^c^
S5 (20/12)	14.4 ± 0.27	84.23 ± 1.07^a^	90.96 ± 3.15^b^	96.7 ± 0.75^c^	98.9 ± 0.35^c^

*C* control, *Prot* proteolytic treatment, *Pect* pectolytic treatment, *T.C* combined treatment

For each sample, the values followed by different letters are significantly different (P = 0.05)

## Influence of pectolytic and proteolytic activities on the content of total polyphenols in oils

The contents of total polyphenols in oils are quite high during ripening and then decrease during the ripening and this whatever the enzyme used (Table 4). Moreover, we find that the proteolytic activities don’t play any significant role in the content of polyphenols in oils when applied alone. The pectolytic activities on them allow a significant improvement of the contents of total polyphenol in oils; this improvement can reach 50 % compared to the control in the early stages of ripening. Contrariwise, when used in combination with the proteolytic enzymes, we find that the wealth of the oil polyphenols increases very significantly and this from veraison to full maturity as it can reach polyphenols gain up to 84 % compared to control. Note that at veraison, oil polyphenols contents can 74 % more compared to the control.

**Table 4:** Effect of pectolytic and proteolytic activities on the total polyphenol content of the oils extracted during ripening

Sample	Content of total polyphenols in the extracted oils (mg/kg)				% de gain en polyphénols totaux par rapport au témoin		
	C	Prot	Pect	C.T	Prot	Pect	C.T
S1 (18/10)	648.84 ± 30.75^a^	701.8 ± 28.5^a^	801.33 ± 32^b^	989.33 ± 29.33^c^	8.7 ± 4.4^a^	23.94 ± 5^b^	53 ± 4.5^c^
S2 (04/11)	459.55 ± 35.4^a^	516.44 ± 27.33^a^	696.44 ± 33.9^b^	800 ± 22.9^c^	12.51 ± 5.9^a^	51.62 ± 7.4^b^	74.3 ± 5^c^
S3 (20/11)	312 ± 38^a^	344.9 ± 40.8^a^	456 ± 26^b^	574.7 ± 32.74^c^	10.56 ± 13.2^a^	46.15 ± 8.4^b^	84.04 ± 10.5^c^
S4 (06/12)	232.9 ± 15.9^a^	249.8 ± 26^a^	347.1 ± 22.32^b^	427.6 ± 24.7^c^	8 ± 10.31^a^	49.03 ± 9.46^b^	83.8 ± 10.4^c^
S5 (20/12)	194.22 ± 14.13^a^	205.33 ± 9.24^a^	248.9 ± 21.7^b^	283.6 ± 16.9^c^	5.36 ± 4.56^a^	27.7 ± 11.25^b^	45.7 ± 8.8^c^

*C* control, *Prot* proteolytic treatment, *Pect* pectolytic treatment, *C.T* combined treatment

For each sample, the values followed by different letters are significantly different (P = 0.05)

## Determination of cell embrittlement olives during ripening

### Evolution of pectins contents and endogenous enzyme activities in olives during ripening

At veraison, the pectins component of the cell walls of olives consist mainly insoluble protopectins. Soluble pectin extracted by water and by sodium oxalate represents a small portion from the whole pectins (Table 5). During the ripening of the drupe, the contents protopectines decrease dramatically and can lose up to 75 % of their initial content. At the same time, there has been a steady increase soluble pectin content so that at maturity, the difference between these two types of pectin becomes very low (Table 5; Fig. 2).

**Table 5:** Influence of proteolytic and pectolytic activities on different fractions of pectin (µg galacturonic acid/g dry matter of olive flesh) contents in olives during ripening

Treatment	Harvest time	Soluble pectins	Protopectins
Control	P1 (18/10)	1805	15,253
	P2 (04/11)	1892	10,240
	P3 (20/11)	2382	8440
	P4 (06/12)	2602	6228
	P5 (20/12)	3072	3807
Proteolytic enzyme	P1 (18/10)	1473	14,653
	P2 (04/11)	1784	9860
	P3 (20/11)	2299	7653
	P4 (06/12)	2352	6107
	P5 (20/12)	2441	4200
Pectolytic enzyme	P1 (18/10)	1520	10,862
	P2 (04/11)	1666	8073
	P3 (20/11)	1712	7110
	P4 (06/12)	2151	5356
	P5 (20/12)	2548	3098
Combined treatment	P1 (18/10)	1321	10,819
	P2 (04/11)	1764	7657
	P3 (20/11)	1823	6341
	P4 (06/12)	2058	5134
	P5 (20/12)	2385	2852

**Fig. 1:**
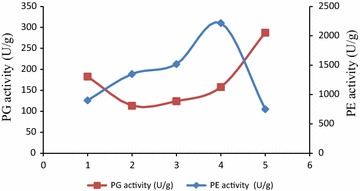
Evolution of endogenous enzymatic activities, polygalacturonases (PG) and pectinestérases activities (PE), during olives ripening

Furthermore, Fig. 1 shows the evolution of enzymatic activities PE and PG of olives during ripening. These two activities generally increase during ripening: PE activities increased gradually to a maximum recorded in the few days before full maturity and then fall again at the end of ripening. PG activity decreased during ripening and then increase slowly at the beginning of ripening, and a larger and faster way to end. The increase in activity was accompanied by a decline in protopectins content and increased of soluble pectins content (Table 5).

### Influence of exogenous pectolytic and proteolytic activities on the degradation of the cell wall during the oil extraction

**Fig. 2:**
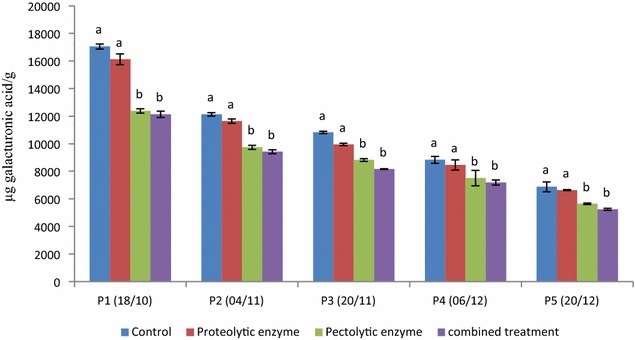
Influence of pectolytic and proteolytic activities on the total pectin content in olives during ripening. Values followed by different letters are significantly different (P = 0.05)

Whatever the enzymatic treatment used, there is still a decline in protopectins content and increased in the contents of soluble pectins during ripening with decrease of the gap between these two types of pectins maturity. This affects the total contents of pectin since protopectins represent almost all pectins at veraison (Fig. 2). In addition, pectolytic activities provide a very significant reduction in the contents of total pectins and this from veraison. However, the application of proteolytic activities alone or added with pectolytic activities implies no significant effect on the degradation of pectic substances and this regardless of the harvest date (Table 5; Fig. 2).

### Highlighting the presence of phenolic compounds bound to the pectocellulosic wall olives

Pectocellulosics cell walls isolated by ethanol precipitation (MIA) were found to contain significant amounts of polyphenols that may be released during the extraction of oil. The enzymatic hydrolysis of the MIA obtained from olives harvested at full maturity with the proteolytic and pectolytic activities used for the extraction of fat (Table 6) shows that the proteolytic activities alone have no influence on this release. However, the pectolytic activities lead to a very significant release of total polyphenols and even in catechin tannin. The combined action of proteolytic and pectolytic activities increases this release.

**Table 6:** Influence of proteolytic and pectolytic activities on the release of MIA polyphenols obtained from olives harvested at full maturity

Treatment	Control	Proteolytic enzyme	Pectolytic enzyme	Combined treatment
[TP] in MIA mg/g of olive flesh	11.9 ± 0.04^a^	12 ± 0.13^a^	13.9 ± 0.2^b^	15.2 ± 0.17^c^
[TC] in MIA mg/g of olive flesh	2.4 ± 0.12^a^	2.54 ± 0.11^a^	3.3 ± 0.01^b^	4.02 ± 0.2^c^

[*TP*] total polyphénol concentration, [*CT*] condensed tannin concentration, *MIA* insoluble material in alcohol

Values followed by different letters are significantly different (P = 0.05)

## Discussion

The ripening of Moroccan Picholine olives variety is accompanied by increased in oil content. These contents do not exceed 15 % at maturity and are quite low compared to the Moroccan varieties that can reach 19, 22 and 23 % respectively of the fresh material for varieties Dahbia, Haouzia and Menara (Hadiddou et al. 2013) and also over to other varieties (Picudo, Picual, Hojiblanca and Frantoio) for which the oil content can reach up to 25 % of plant fresh weight (Gomez-González et al. 2011; Jiménez et al. 2013; Beltran et al. 2004). The same findings were obtained in Moroccan olive groves where farmers get an oil yield situated between 13 and 18 %. This yield is affected by the farming techniques and the date of olive harvest (Ait Yacine 2002). In addition, the water content of the olives is an important parameter that determines the percentage of the oil during the extraction. During ripening, the humidity decreases with increasing oil content in the olives. In our case the low content of oil in the olives may be due to high humidity during the last sampling.

The extractability of the fat during the ripening is to bind with their location in the cell and with the condition of the fragility of fruit tissue. The location of the oils in fruits has been the subject of several studies: the oils accumulate in the cytoplasm of olives cell (Rangel et al. 1997) primarily in the mesocarp, but also in the endocarp and epicarp. These oils a rein particular localized in the vacuoles of the cells in free form and in the cytoplasm in bound form (Ranalli et al. 2001). Therefore, the cell walls, the cell membranes and the vacuolar membranes play a role like a physical barrier preventing the extraction of the cell contents, and their degradation can improve the extractability of the fat. Moreover, the state of cell fragility is estimated by the protopectines contents that decrease on a regular basis during the ripening to very low values at maturity. The work of Mínguez-Mosquera Isabel et al. (2002) on the olives cv. Hojiblanca disagree with that study; for these olives, protopectins rate remains almost stable during the ripening while the soluble pectin rates decrease. However, these results confirm those found by Amrani Joutei et al. (1994) on grapes. The degradation of pectins is itself related to the contents of pectolytic enzyme activities in the fruit. These latter act on the protopectins (soluble fractions NaOH and HCl) to degrade into soluble substances in water. This solubilization can be completed by other activities such as cellulases and hemicellulases activities. This water-soluble fraction has many methyl esters will be hydrolysed by the pectinestérases giving soluble substances oxalate there fore relatively poor in methyl esters. In these conditions, polygalacturonase are involved and degrade these molecules into smaller chains of galacturonic acids. The result is a significant release of cell contents from about 25 % at veraison to 84 % at maturity. Nevertheless, the evolution of the cell fragility in olives tissues do not get a oils rich of polyphenols, since we find that the contents of these molecules decrease in a very significant way to maturity. During fruit ripening, there is the modification of the phenolic profile. In the early stages of growth, oleuropein is the major phenolic compound of the fruit; its concentration can reach up to 14 % of net weight (Amiot et al. 1986). Then, its concentration decreases very significantly from veraison due to the presence of anthocyanins (Bianco et al. 1999; Servili et al. 2004; Gόmez-Rico et al. 2009). This steady decline in content oleuropein present in the fruit is due to the β-glucosidase activity, released into the paste during grinding cause degradation of phenolic groups, leading to their transformation into oleuropein aglycone and 3,4-DHPEA-EDA. This reduction may also be due to activation of the polyphenoloxidase (PPO) and Peroxidase (POD) during the formation of the olive paste subjected to mixing (Bianco et al. 2001; Gόmez-Rico et al. 2009).

Furthermore, a structural modification of the phenolic compounds during the ripening or during the extraction of oils may also have an influence on the dissolution of these compounds in the oil (Artrajo et al. 2006). In fact, some studies realized on the localization of phenolic compounds in olives (Uccella 2001) and grapes (Amrani Joutei et al. 1994) showed that phenolic compounds catechin tannins kind are located mainly in the vacuoles in free form or bound to tonoplast, but also in a form bound to the cell walls of the fruit. These phenolic compounds can also condense in vacuoles during the ripening making them more difficult to remove. There by release requests the intervention of external factors such as the use of enzymatic activities for the degradation of cell walls and membranes which constitute a barrier to the diffusion of cell contents.

Total pectin contents know a significant decrease during the ripening of the olives. This decrease is closely related to the endogenous enzyme activities of the fruit of the olive tree, PG and PE activities, provides softening of the drupe. Based on the work of Brady et al. (1982) and Dellapenna et al. (1990) on tomato and those of Pathak and Sanwal (1998) on bananas during ripening, Mínguez-Mosquera Isabel et al. (2002) concluded that the accumulation of PG activity in the first stages of ripening is endo-PG type which is sufficient for the depolymerization of the pectin chain in vivo. Then an exo-PG (PG 2) and endo-PG (PG 3) accumulate during maturation.

The moderate decline in total pectin contents recorded in the middle of the ripening (3rd and 4th sampling), is directly related to high values of the activity of PE in fruits and low activity in PG. This may promote calcium bridges formation in pectin chains and therefore smaller losses of the total pectin content (Mínguez-Mosquera 2002; Alonso et al. 1995; Ketsa et al. 1998).

At the end of ripening, the PG activity peaks and degradation of the pectic material increases but at a lesser level than that detect early ripening since the fruit reaches its full maturity. Heredia et al. (1993) proposed that the PG must be associated in this step by glycosidase type activities.

The application of proteolytic activities does not have any significant role in the degradation of cell walls and this whatever the harvest date. Contrariwise, the application of pectolytic activities on olives allow a very significant degradation of olives tissue structures with a decrease in protopectins levels from veraison to maturity and these levels are lower than those of the control mainly when these pectolytic activities act in combination with the proteolytic activities. Under these conditions, the use of these activities results in a weakening of the parietal cell and membrane structures allowing the release of a larger amount of the cell contents. These observations are also confirmed by the liberation and enrichment of oil with phenolic compounds. In fact, in the absence of any enzymatic activity, the contents of phenolic compounds in oils decrease about 68 % during the maturation, while the contents in olives only decreased by 10–20 % which confirms the hypothesis of the presence of a physical barrier preventing their release. This hypothesis, which states a bond of polyphenols with polysaccharides of the cell walls and the component proteins of vacuolar membranes, is confirmed by the use of pectolytic and proteolytic activities: If the proteolytic activities alone have no influence on the release of phenolic compounds from olives, the pectolytic activities alone and especially in combination with the proteolytic activities allow to enrich the oils with these compounds and this from veraison. The levels of oils enrichment with phenolic compounds by the combination of exogenous proteolytic and pectolytic activities increase during the first stages of ripening when the cell walls of olives are not completely weakened by endogenous activities then these levels decrease towards the end of ripening when the parietal structures are naturally degraded by endogenous activities. Under these conditions, the hypothesis of the presence of a mechanical barrier which opposes the release of phenolic compounds in the oils is verified: we can conclude that the pectolytic activities degrade the parietal structures releasing the polyphenols related to parietal structures but also the polyphenols that are released by the degradation of the component proteins of vacuolar membranes. The use of proteolytic and pectolytic activities on the insoluble material alcohol consisting mainly of pectocellulosic structures of olives cell walls confirms the hypothesis of the presence of polyphenols bound to the cell walls of the fruit, but also to the vacuolar membranes making their release difficult during the extraction of oil. The release of these polyphenols in the medium is made possible by the degradation of the parietal and membrane structures of the cells by enzymatic activities. These results are in agreement with those of Vierhuis et al. (2001) who have shown that the use of enzyme preparations leads to an increase of the levels of derivatives secoiridiode as the aldehyde form of elenolic acid linked to 3,4-dihydroxyphenyl ethanol (3,4-DHPEA-EDA) and the isomer of oleuropein aglycone (3,4-DHPEA-EA) in olive oil and also confirm the results obtained from the grapes that show an increase in levels of tannins during red winemaking when the enzyme activities are added to the vintage (Amrani Joutei and Glories 1995).

## Conclusion

The role of the proteolytic activities in combination with pectolytic activity was studied on the release of the fat and phenolic compounds during the extraction of olive oil. In this study, we were able to highlight the presence of phenolic compounds linked to difficult releasable parietal structures during oil extraction. Furthermore, we showed that the application of proteolytic activities alone are ineffective on extractability of the cell contents, while their use with the presence of pectolytic activity causes an increase in the diffusion of the fat and it is wealthy on phenolic compounds mainly when olives have not reached sufficient maturity. The action of these activities lies in the fact that firstly the proteolytic activities degrade the component proteins of membrane structures releasing the polyphenols associated with these structures and promotes the diffusion of the oil, on the other hand, the pectolytic activities degrade the parietal structures allowing the passage of the cell contents and releasing the same time the polyphenols associated with cell walls that can enrich the phenolic content of oil.
